# Visualizing Physical Activity Patterns among Community-Dwelling Older Adults: A Pilot Study

**DOI:** 10.3390/sports6040135

**Published:** 2018-10-30

**Authors:** Monika Haga, Katerina Vrotsou, Ebba Bredland

**Affiliations:** 1 Department of Neuromedicine and Movement Science, Norwegian University of Science and Technology, 7491 Trondheim, Norway; ebba.bredland@ntnu.no; 2 Department of Science and Technology, Linköping University, 581 83 Linköping, Sweden; katerina.vrotsou@liu.se

**Keywords:** time-geographic perspective, visualization, physical activity patterns, energy expenditure

## Abstract

Regular physical activity relates to physical and mental functioning in older people, and promoting physical activity has the potential to substantially reduce functional decline and improve well-being. Despite this, investigations of the physical activity quotient through participation in functional activities in everyday life have traditionally gained limited focus among older populations compared to leisure-time physical activity and exercise. Considering the accumulated evidence of the health benefits of low-intensity physical activity, exploring and measuring such activities in this population is highly relevant. The aim of this study was to visualize and describe older people’s physical activity patterns in daily life using a time-geographic approach in combination with the estimation of metabolic equivalents (METS). To exemplify the new method, a sample of nine retired men (65–82 years old, mean age 76.4 ± 5.8) with no homecare services from the municipality was recruited. In order to enable a visual analysis of the physical activity patterns in daily life, we developed the VISUAL-PA software, which is a visual analysis tool that includes METS to account for intensity and enables the analysis of distinct types and domains of physical activity. The VISUAL-PA software creates graphic outputs of physical activity patterns that enable the identification, visualization, and analysis of distinct types and intensities of physical activity in addition to sedentary behavior. The use of VISUAL-PA can contribute to a broader understanding of the complexity in physical activity patterns among older adults in terms of dimensions such as activity patterns and habits, domains, and intensity level. To strengthen the public health strategies that promote health and an active lifestyle, additional knowledge about physical activity patterns is necessary. Moreover, the visualization of physical activity can enable reflections on and awareness of activity habits and preferences, and thus facilitate behavior changes in older individuals.

## 1. Introduction

An aging population will have a major impact on the delivery of health care and contribute importantly to the rise in health care costs in the following years. To address these challenges, we need to develop better and more sustainable interventions and policies optimizing physical and psychosocial functioning. Regular physical activity relates to physical and mental functioning in older people [1,2], and promoting physical activity has the potential to substantially reduce functional decline and improve well-being [3]. Even so, the transition into retirement and older age is associated with engagement in lower levels of physical activity and more time spent in sedentary behavior [4]. An essential challenge that must be addressed is the new trend of increased inactivity in everyday life, including prolonged sitting time [5,6]. Health authorities in Norway have encouraged municipalities to spread health promotion knowledge to older adults by doing home visits, where information about physical activity is an important issue [7]. This in turn requires new knowledge about healthy older adult’s physical activity patterns and the development of innovative health promotion initiatives to prevent disease and optimize well-being [8].

Physical activity is a complex, multidimensional behavior in terms of activity patterns, habits, and levels [9] that encompasses dimensions such as the purpose, context, and intensity level of the activity [10,11]. Physical activity can be divided into unstructured activity incorporated in daily life—*non-exercise physical activity* (NEPA) [12]—and intentional exercise, which includes structured, planned, and repetitive activities. NEPA are functional activities that are performed in daily life (such as carrying groceries, vacuuming, or walking to stores) of various intensity that represent important qualitative health-promoting activities in older people [13,14]. There are arguments that older adults may find it difficult to meet the current activity recommendations of moderate and vigorous intensity, and that increased light intensity could be a potential factor to health promotion by reducing sedentary time and paving the way to more intense activities [15,16,17]. Functional everyday activities are often compound motor actions that require movement competence such as manual dexterity, balance, and coordination, which are skills that are important to function in everyday life [18]. However, investigations of the physical activity quotient through participation in such ordinary activities have traditionally gained limited focus among older populations compared to leisure-time physical activity and exercise [17,19].

Physical activity questionnaires and accelerometers have been extensively used to determine activity levels, many of them derived from an energy expenditure-based perspective, mainly measuring activities of moderate and vigorous intensity [11]. These methodologies have some important limitations. For example, physical activity recall is associated with a significant overestimation of physical activity, and accelerometers generally underestimate physical activity because they are unable to quantify activities such as cycling, swimming, and upper-body activities [20]. In addition, the outcomes of such studies are difficult to translate into individual or policy recommendation other than enhancing physical activity behavior.

Moreover, assessing activity based only on intensity level does not reflect the actual PA patterns and behavior of older people, because they perform more functional everyday activities. In this context, examining PA domains such as recreation, transport, self-care, and household care are recommended [11]. This broad spectrum of physical activity domains reflects the high variability in the physical activity behavior in the elderly, and underlines the necessity of assessing other relevant aspects beyond PA intensity and energy expenditure [10]. Future research should add a biopsychosocial perspective to the existing energy-based approaches [10] by using assessment tools that reflect PA patterns and context, including NEPA. Assessing health-promoting functional activities of low intensity, and putting demands on manual dexterity, balance, and coordination, (such as NEPA) as well as sedentary behavior across PA domains, becomes important [11]. The time-geography diary (TGD) approach gives information about the PA domains, and will provide a deeper knowledge about what kind of activities are important to older adults and how activities of everyday life facilitate or constrict a physically active lifestyle. Assessment tools that reflect these aspects could increase our conceptual framework to physical activity in older people.

The process of changing health behavior is dependent on individual choices based on motivations and confidence in one’s own ability to make these changes [21]. Therefore, scientific approaches and methodological development that could raise consciousness and awareness about physical activity habits and preferences at the individual level are required. Knowledge about how patterns of daily occupation and activities develop, as well as the environments preceding activity patterns in daily life, is essential to enhance lifestyle changes [22].

The time-geographical approach [23] is facilitated through the time-geography diary (TGD) method [24]. The diary method has been used in different studies in health and social sciences [22,25], and is also considered to be a method that may enable a deeper understanding of occupational engagement in community-dwelling older people, including physical activity [26]. Furthermore, the TGD method is found to be useful in client-centered interventions, as it may facilitate reflections on changes in the patterns of daily activities [22]. Time geography is a framework for describing reality and a notation system for representing individuals’ movement and activity in time and space [23]. The TGD method provides an effective approach to collecting data that enables the systematic study of activities of everyday life. The method focuses on what people do and how everyday life is shaped by which activities an individual prefers and chooses for engagement. It illustrates routines and chosen activities in a social and geographical context. To visualize the activity patterns, the diary data can be coded and entered into a time-geographic visualization software program [24,27].

The program makes it possible to illustrate the sequences of activities performed as an activity path (graphs), which is a graphic output that the users writing the diaries easily can recognize as their everyday activity pattern [28]. The visualization of information and data may increase perceptibility and reveal patterns within complex information [29]. In this way, the description of everyday life may be used as a starting point for a dialogue about patterns and habits of activities [30].

Time-use perspectives represents a promising approach in the study of physical activity and energy expenditure [31,32,33], especially as it appears to be more valid for non-occupational populations surveillance compared to more traditional self-reported assessments [34]. The Ainsworth Compendium of Physical Activities [35] is a well-known and documented approach to allocate metabolic equivalents (METS) to different types of activities. Time-use data can be used to study physical activity energy expenditure by assigning MET values to daily activities using the compendium [31]. The compendium is used globally to quantify the energy cost of PA in adults for surveillance activities, research studies, and, in clinical settings, to write PA recommendations and assess energy expenditure in individuals. Thus, it is a living document that is proceeding in the direction of being entirely evidence-based [35]. Earlier research has linked the TGD approach with metabolic equivalents (METS), relying on the Compendium of Physical Activities [35] to study physical activity patterns in daily life in older men [36]. The results showed that some men demonstrated a high level of physical activity without performing any intentional exercise, as their activity habits and patterns in daily life were linked to engagement in NEPA. Conversely, although some participants reported that they exercised regularly three times a week, much of the remaining time was spent on sedentary activities (e.g., TV viewing, reading), and almost no NEPA was reported. Older adults seem to have different opportunities, needs, and habits with respect to performing physical activity in their daily routine. In order to design and develop health-promoting actions to suggest to the elderly by health care personnel at the local community level, these considerations must be taken into account.

In this pilot study, time-use data was used to study physical activity energy expenditure by assigning MET values to different types of daily activities using the Ainsworth Compendium of Physical Activities [35]. According to previous research, the TGD method in combination with METS may enable the analysis of physical activity and sedentary behavior in daily life in older adults [36]. To enable the visual analysis of event sequence data such as activity diaries [27,37], we developed the VISUAL-PA software, which is a visual analysis tool. This tool is a modified version of the time geographic software program VARDAGEN that was introduced by Ellegård [24] and the software program VISUAL-TimePAcTS [27,37].

VISUAL-PA includes METS to account for intensity, and creates graphic outputs of physical activity patterns that enable the identification, visualization, and analysis of distinct types (e.g., NEPA/intentional exercise) and intensities (light, moderate, vigorous) of physical activity in addition to sedentary behavior. The aim of this study was to visualize and describe older people’s physical activity patterns in daily life through applying a TGD approach, in combination with the estimation of METS, and discuss how this approach could deepen our knowledge of the physical activity patterns in everyday life.

## 2. Methods

### 2.1. Context of the Study

This pilot study was originally derived from a health promotion project for community-dwelling older people to promote active aging [36]. The health promotion project focused on the everyday physical activity patterns of the elderly to analyze and evaluate physical activity and sedentary activities as a basis for assisting the process of changing physical activity behavior. The time-geographic perspective was adopted, and these notions were extended to include metabolic equivalents (METS) associated with the performed daily activities.

### 2.2. Participants

Participants that were enrolled in the original health promotion project for community-dwelling older adults were included in this pilot study. Participants were recruited through the convenience sampling strategy, as they were asked to participate by the “Info center for seniors”, which is an information office for promoting health in retired people. Handouts with written information were distributed. The inclusion criteria consisted of retired men aged 65 and over with no homecare services from the municipality, and who were capable of and interested in diary-writing for one week. In order to gather data that could provide a wide range of information about the physical activity patterns of daily life, variation in age, living conditions, and co-habitation was taken into consideration. In addition, men from different activity groups [36] were included. No health information was registered (i.e., diagnoses or functional status). Nine men aged 66–83 years (mean age 76.4 ± 5.8), completed a diary over the course of a full week each. Thus, a total of 63 completed daily diaries were made available for this study.

### 2.3. Procedure

#### Diary data

The method we propose relies on a TGD notebook (open diaries), as recommended by Ellegård [24]. Individuals were instructed to take notes every time they started a new activity throughout the course of the day, from getting out of bed in the morning until going to bed at night. The headlines in the diary (i.e., the variables to be collected) include Time, What I Do (activity), Where I Am (location), Who I Am With (socialization), and Comments. The diary can be maintained for a few days, but to capture the rhythm of the week and the differences between physical activity on weekdays and weekends [38], the participants registered their activities over one ordinary week. The data collection was carried out over late autumn and the start of winter, and the participants had about six weeks to find a suitable time (an ordinary week) before handing in the notebook. The last author met the participants before they started writing to give information, encourage them to write comments, and answer any questions. This author also met them after the diaries had been written to ensure the week was an ordinary week, and to talk about their experience and any additional comments. No participants missed a day of input.

## 3. Data Analysis

### Activity Categorization

To represent and compare the collected data, the written diaries were coded in accordance with the time geographic scheme, by the first and last author. Each occupation was given a unique code, and then entered into the time-geographic program VISUAL-PA. In this study, we adopted a modified version of the hierarchical activity categorization scheme introduced by Ellegård [24]. This categorization scheme has five levels of hierarchy, which are referred to below as “levels of detail” (LOD). Each activity description at LOD n+1 is broken down into a more detailed description at LOD n; LOD 5 is the most general level, and LOD 1 is the most detailed one. For example, “Transportation” at LOD 5 is broken down to “travel by car”, “travel by foot”, “travel by bicycle”, etc., at LOD 4. At the most general description level (LOD 5), there are eight main activity categories: (1) self-care; (2) care for others; (3) household care; (4) recreation; (5) transportation; (6) food-related activities; (7) intentional exercise (IEX); and (8) work and school.

The new categorization scheme in VISUAL-PA has two primary differences compared to the original TGD one. First, in the new categorization scheme, intentional exercise has been added as a separate category, and has been enriched with several new activities, including resistance (weight) training, calisthenics, jogging, swimming, bicycling, team exercise, etc. Second, in order to investigate the physical activity quotient through participation in ordinary daily activities, additional information has been associated with the activities in the categorization scheme. Three additional variables have been added to each activity description: (1) whether the activity is NEPA; (2) whether it is intentional exercise; and (3) the METS value corresponding to the activity assigned by the Compendium of Physical Activity [35]. Associating activities with NEPA (such as garden work, preparing food, and cleaning the house) makes it possible to differentiate between different types of physical activity (intentional exercise versus NEPA) and determine when, for how long, and in which of the main categories (e.g., transportation and household care) NEPA is performed in daily life (Table 1).

Assigning METS values to activities enables the identification of sedentary behavior, the quantification of the intensities of the activities, and a comparison between different types of physical activity. METS are used to classify activities based on the rate of energy expenditure and intensity of the activity [35], and make it possible to group the activities into sedentary (<1.5 MET), light (1.5–2.9 METS), and moderate/vigorous (>3 METS) intensities. Since each activity in the categorization scheme is associated with a MET value, it is possible to color code the activities with respect to their physical activity intensity level classification (sedentary activity, light intensity, and moderate/vigorous intensities) (Table 1). VISUAL-PA permits a differentiation between moderate/vigorous intensities belonging to NEPA or intentional exercise. The physical activity intensity level is represented by the following colors in VISUAL-PA: sedentary activity is red, light intensity NEPA is yellow, moderate/vigorous intensity NEPA is dark green, and intentional exercise of moderate/vigorous intensity is light green. In VISUAL-PA, sedentary activities and NEPA are coded under the same main category. For example, “Walking to the city” (NEPA) and “Sitting on the bus” (sedentary behavior) are both coded under “Transportation”. Therefore, by coding the activities according to METS, it is possible to sort out NEPA from the diary data.

## 4. Results

This section will describe how the collected data are visually represented and studied using VISUAL-PA. Physical activity pattern diary representation of older individuals will be viewed, and examples of diary visualizations from our sample will be presented.

**Figure 1:**
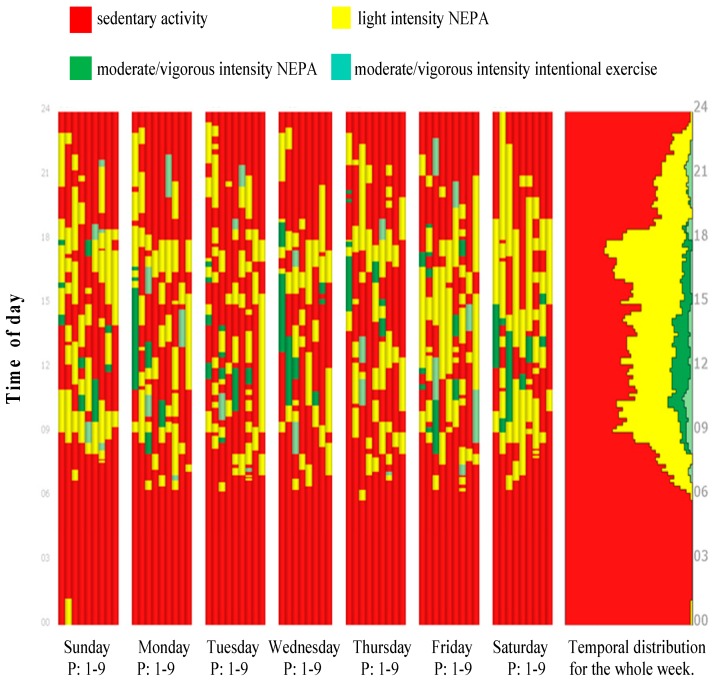
Visualization of the nine participant´s (P) diaries from Sunday to Saturday. Time is represented on the vertical axis. The activities are colored with respect to the type of physical activity and intensity level and sedentary activity. Right panel shows a temporal distribution of physical activity for all individuals for the whole week.

**Figure 2:**
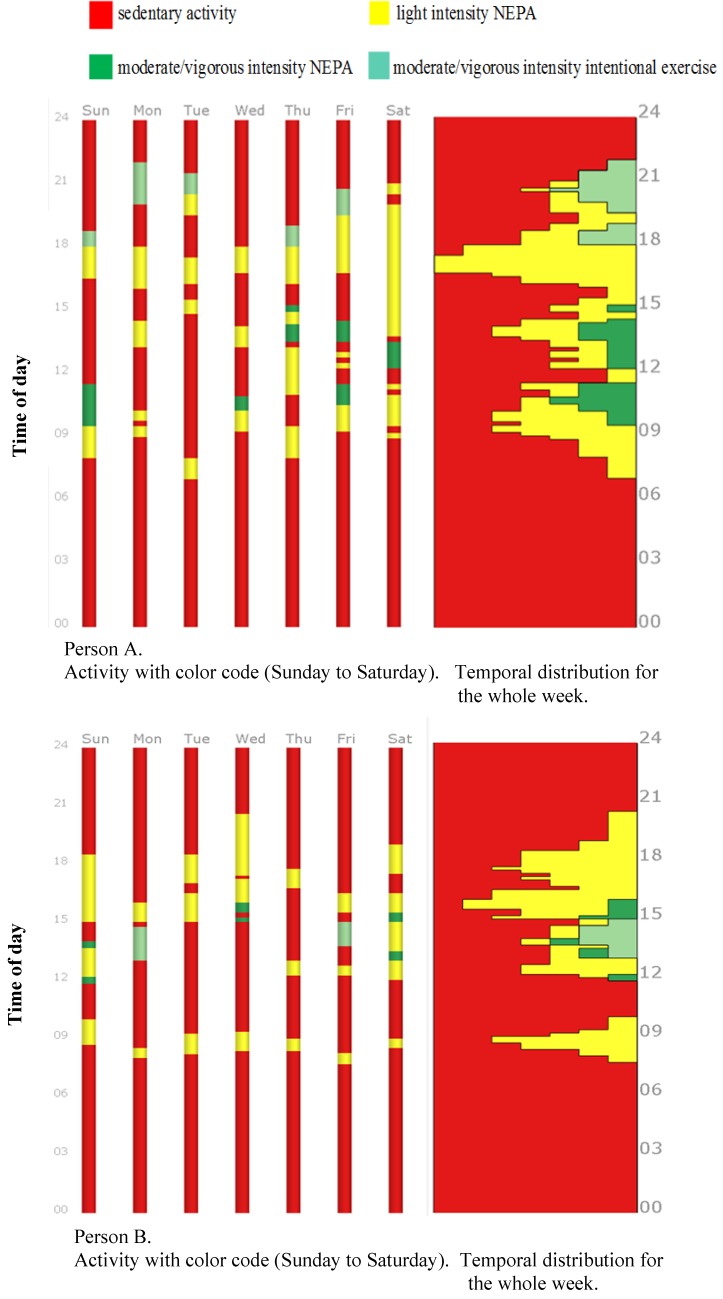
Visualization of the activity sequences of person A and B for all days during a week. Time is represented on the vertical axis. The activities are colored with respect to the type of physical activity and intensity level and sedentary activity. Right panel shows a temporal distribution of activities for the whole week.

Once the diary data were coded according to the TGD activity hierarchy scheme, they were imported and visualized in VISUAL-PA. The central representation used within VISUAL-PA is the individual path inspired by the time-geographical approach and notation [23]. Individual paths represent the sequence of activities that individuals performed during the day. Two different views of individuals’ paths are available in VISUAL-PA: the front and the side view. The front view is appropriate for displaying an individual’s diaries for all of the days in a week or several individuals’ diaries for each day to compare their days (Figure 1 and Figure 2). In the front view, diaries resemble stacked bar charts within a coordinate system in which individuals are represented on the horizontal axis, and time is represented on the vertical axis going upward. Individuals’ diaries can be sorted along the horizontal axis with respect to several variables such as age, sex, and day of the week.

Figure 1 shows all of the nine studied individuals’ diaries colored with respect to the type of physical activity and intensity level (presented in the front view). A frequency graph showing the temporal distribution of physical activity for all of the individuals during the week is shown in the right panel of Figure 1. This representation reveals the temporal distribution of physical activity types. At the group level, a relatively high amount of time is spent in low intensity NEPA (yellow color), and this type of activity is distributed across the whole day. More time is spent in moderate/vigorous intensity NEPA (dark green) compared to intentional exercise (light green). While moderate/vigorous intensity NEPA is mainly executed during the daytime, intentional exercise is an activity that is performed both in the morning and the evening. Sedentary activity is more common in the evening, as one can see a marked decline in PA level after 18:00. Finally, as an extension of VISUAL-PA, there is functionality in VISUAL-PA to automatically identify sequences of activities as frequent patterns in the data, which makes it possible to explore common activity patterns among individuals [39].

Details about individuals and their diary data can be retrieved by clicking on the bars representing the activities in the visualization. When clicking on an activity in the visualization, details about the time, type of activity, and corresponding METS value are shown in a pop-up tool tip, and background information about the individual performing the activity is displayed in the user interface of VISUAL-PA. Activities of interest can be highlighted in the system to more closely study their distribution across the diaries. Furthermore, VISUAL-PA makes it possible to add up the amount of time devoted to physical activity and sedentary activities and produce frequency tables as well as graphs showing the total daily engagement in physical activity and sedentary activity at the individual or group level.

In Figure 2, two individuals’ physical activity patterns over the course of an entire week are displayed (A and B). To the left, the activity sequences are shown colored by intensity level of physical activity and ordered along the horizontal axis by day of the week, from Sunday to Saturday. To the right of the figure, a frequency graph is displayed showing the temporal distribution of physical activity over the course of the day for the entire week for person A and person B, respectively. For person A, the week is more colorful, which implies the participation in many categorized activities, such as NEPA and intentional exercise (more yellow and green) compared to person B. Person A reports continuing his activities later in the evenings compared with person B, who mainly exhibits sedentary behavior after 18:00. During the whole week, person A spent 33 h and 45 min in light intensity NEPA (1.5–2.9 METS), 7 h and 5 min in moderate/vigorous intensity NEPA (>3 METS), and 6 h in intentional exercise. Person B spent 27 h and 20 min in light intensity NEPA (1.5–2.9 METS), 2 h and 25 min in moderate/vigorous intensity NEPA (>3 METS), and 3 h in intentional exercise.

**Figure 3:**
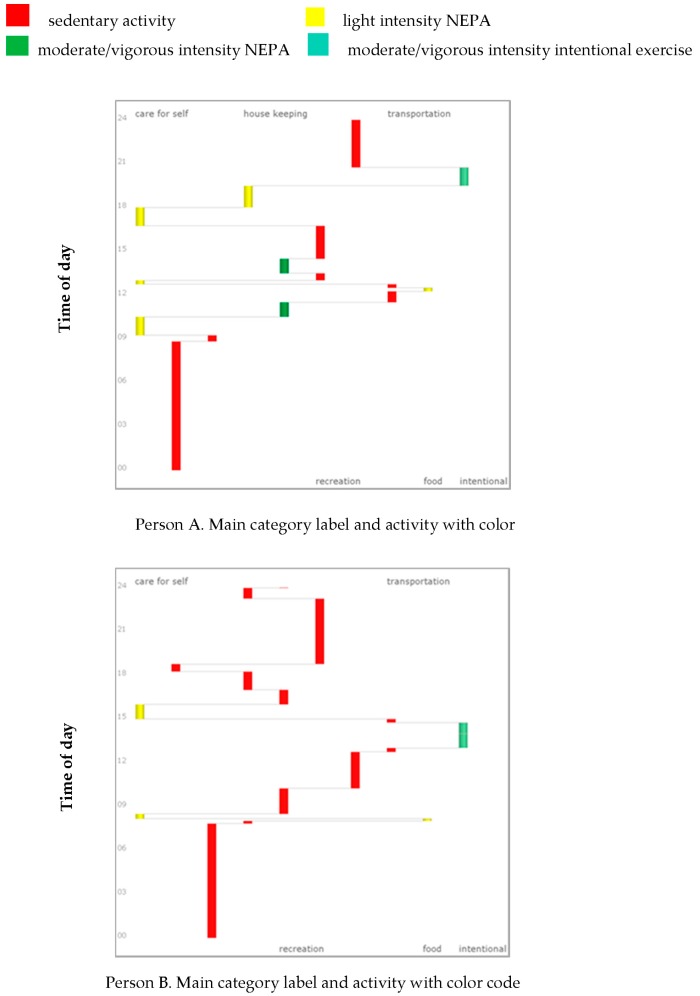
Side view visualization of the activity sequences of person A and B in VISUAL-PA colored with respect to the physical activity intensity level of the activities they are performing during one day. Time is represented on the vertical axis.

The side view is appropriate for viewing single individual paths and studying the activities that they perform in more detail (Figure 3). In this view, the horizontal axis shows the intensity levels and physical activity type (intentional exercise and NEPA), and time is again represented on the vertical axis going upward. The individual path is represented as a continuous trajectory moving from one activity to the next during the day, and should be read from the bottom to the top. In Figure 3, graphs to visualize the diary of one of the days reported by two different individuals in the dataset, A and B, are presented. The visualizations represent the daily activity sequences of the individuals, including intensity levels and physical activity type (intentional exercise and NEPA). The individual shown in graph A is an active man who is 73 years old, and living in a house with his wife. He is doing physical work every day, such as maintaining the house or doing voluntary work outside. His activities fell into many of the main categories: self-care, household activities, recreation, and transportation, in addition to intentional exercise. The person shown in graph B is an 82-year-old man living in an apartment with his wife who is doing all of the housework. His hobby is to write. He exercises and is engaged only a small amount of light intensity NEPA in everyday life. His activities fall into the main categories of self-care, recreation, transportation, and intentional exercise. A high proportion of red color in the activity graph implies that a person exhibits sedentary behavior throughout the day. The visualization of person A shows a variety of yellow and green colors, and only extended periods of red during the night. Person B, on the other hand, displays many prolonged periods of red during the day. Even though this individual is executing some intentional exercise, as recommended, the rest of his daily activities can be categorized as sedentary.

## 5. Discussion

The purpose of this study was to identify, visualize and describe older people’s physical activity patterns in daily life using a TGD approach, and explore some of the benefits of using such a method. VISUAL-PA offers significant useful functionality for visually analyzing the collected diaries and the different types of physical activity that are present in them. This paper demonstrates how the use of VISUAL-PA can contribute to a broader understanding of the complexity in physical activity patterns among older adults in terms of dimensions such as activity patterns and habits, domains, and intensity level.

Engagement in NEPA and intentional exercise daily is not only dependent on capacity and function, it is also influenced by context [40]. However, an important limitation of the current literature is that most studies have only examined energy expenditure and time use with little reference to the activities and the contexts in which individuals are engaged. This situation is true for activities of daily living. Information on the amount of time spent in different physical activities is incomplete in relation to explaining how and why people use their time in a specific way [22]. Hence, using assessment tools such as VISUAL-PA that reflect an individual’s physical activity patterns in a context could be beneficial.

Both individual and environmental determinants, such as relationship status and physical context, influence physical activity patterns [41]. However, relatively little is known about the association between variables such as gender, socioeconomic status, geographical location, housing, and seasonality, and individual physical activity patterns and intensities, including NEPA in the older population. Therefore, the TGD approach may contribute to a deeper understanding of the interaction between activities and context [26]. Bredland et al. [36] found that men living alone or with a sick spouse tended to perform a relatively large amount of NEPA, but engaged in little or no intentional exercise. Moreover, when participants moved to a new apartment with a spouse, they tended to report performing less NEPA. Moving to an apartment may constrain activities differently that living in a detached house or a townhouse (e.g., there might be no stairs in the apartment, there might be easy access to elevators, there might be reduced possibilities for gardening or snow shoveling). Measuring involvement in NEPA may be especially important because different physical environmental characteristics relate to specific physical activity domains such as transportation, recreation, and household care [40], as illustrated in Figure 3. In this way, the approach can be helpful to facilitate older individuals to reflect on how their social and physical context influences their own activity opportunities and choices. Information about diagnosis and functional status were not registered in the study. In this context, it is likely to assume that some of the participants had some health conditions or symptoms that could have influenced physical activity patterns, such as pain, vision problems, arthritis, and hypertension.

The TGD method contributes by adding a biopsychosocial perspective to existing energy-based approaches [11] that assesses and evaluates physical activity in a broad sense, including different types and ranges of such activities. Individuals that display inactive lifestyles will often be recommended to start in different organized activity groups in the community [36]. However, suggesting exercises or participation in physical activity groups to people who have never exercised before is not facilitating user involvement, and can cause low compliance. Contrary, by analyzing their everyday routines using the TGD method, this could provide an opportunity for individuals to reflect on their own physical activity engagement and ensure subjectively important occupations [26], which is an important aspect in healthy aging. It is probable that engaging in daily activities that are meaningful and adapted to the user’s contextual aspects could endorse increased activity levels [42]. Considering the accumulated evidence of the health benefits of low-intensity PA [43,44,45], reducing sedentary time, and increasing the physical activity level to even light intensities offers health benefits [15,46]. Walking to the shop instead of taking the car or taking the stairs instead of an elevator could be small but important adjustments [36].

Better knowledge of how older individuals participate in activities and physical activity in daily life can improve the development of actions enhancing health promotion and an active lifestyle. The highlighting of physical activity patterns using geographic diaries and visualization (as shown in Figure 2 and Figure 3) can facilitate reflections on activity habits and preferences in everyday life [22,30]. The visualized information can be considered valid, since it emanates from their own reported activities [47]. Looking at the visualization of person B in Figure 2, there are long periods of red, which indicate sedentary behavior. It is especially interesting to see that there are long spans in the evenings after roughly 18:00 without any physical activity; he reports that this is time spent watching TV. In a clinical setting, this information can be used to understand and increase awareness of one’s own physical activity patterns, and can be used as a common starting point for discussions related to everyday routines between the individual and professional. Visualizing physical activity habits can be a good starting point for discussions with seniors about change habits and promoting physical activity in everyday life. Visualizing habits can help focus on resources within an individual’s control and the limitations associated with utilizing those resources [30]. Changing activity behavior is a dynamic process, and any behavior change intervention should enable individual consciousness regarding the specific barriers and resources influencing their physical activity patterns [21]. In combination with stimulated recall interviews and motivational interviews, the approach can be used as a therapeutic and pedagogical tool in physical activity interventions, and it can clarify and enhance the processes of goal-setting and an evaluation of goal achievement [22]. This new way of analyzing and evaluating physical activity behavior in older people can enable user involvement and strengthen motivation, coping, and empowerment among seniors related to healthy and active aging. Encouraging and supporting individuals to make physical activity lifestyle changes can be set out in an individual-centered setting, as well as in group settings. Peer support groups could be established under the supervision of a professional or trained older individuals.

The TGD method is both appropriate to understand behavioral change at the individual level and at the group level [26]. When planning and developing community actions tailored to community-dwelling older adults, knowledge derived from such an approach could ensure their needs and motivations. Better knowledge of possible moderators (e.g., gender, housing, and seasonality) of physical activity patterns could facilitate the development of community actions and population-specific recommendations and strategies [48]. In a research setting, this method could be used to explore the patterns of PA in daily life (NEPA and IEX) and the sedentary behavior of older adults, the influence of the moderator variables such as gender, socioeconomic status, geographical location, and seasonality, and their relationship to psychological well-being and physical functioning. Combined with a qualitative approach that includes methods such as interviews and focus groups, this method could also help develop a clearer understanding of the barriers and facilitators of PA and sedentary behavior in daily life [42].

Health-promotion strategies focus on encouraging a population to develop its own resources and expertise to achieve the best possible health and quality of life. These strategies highlight that the responsibility for health lies both within the individual (i.e., on a personal level) and at social and environmental levels. To make healthy lifestyle choices requires users to receive adequate information, guidance, and training so that they can increase their health literacy [49]. To strengthen strategic community actions enhancing health promotion and an active lifestyle, additional knowledge about physical activity patterns among the older population is necessary. From a health-promoting perspective, a focus on facilitating and maintaining functional activities that are familiar and meaningful to older adults may be more effective than attempting to establish new habits related to organized exercise programs to increase physical activity behavior [36]. Functional activities in everyday life become more relevant than other activities (e.g., sports activities) when we age, because those activities maintain an individual’s independence and well-being [46]. Therefore, using this approach can facilitate the development of interventions that are tailored to both local and individual needs that fit around motivation processes and current behavioral habits, and take environmental constraints into consideration.

### Limitations

A limitation to using TGD in data collection may be self-registration: participants can be influenced by the ways in which they wish to present themselves, as well as assumptions about what they are expected to write down. Furthermore, the precision of the findings depends on the detail of the diary entries, which differs between people. Also, it might be a limitation that the TGD method registers only one activity at a time, as participants sometimes reported that they were occupied with parallel activities in the diary [26]. When analyzing the diaries, the researcher must approximate the time used on each of these two activities, which may be slightly incorrect [36]. Applying standard METS values to determine the energy cost of physical activity within individuals, and especially in older individuals, could also have some limitations [35], and future research should explore the relation between the TGD method and accelerometer-obtained PA and sedentary patterns in older adults. However, being able to identify and visualize light-intensity physical activity in the everyday life context may be a critical issue, because spending time engaging in light-intensity, functional activities reduces sedentary time and preserves functionality in older adults [12].

## 6. Conclusions

VISUAL-PA can help us acquire a broader understanding of the complexity of physical activity patterns in older adults and contribute to an improved conceptual physical activity framework in older people. Obtaining accurate estimates of activities are challenging; however, this approach can be considered as a methodological and pragmatic input to future time-use research on physical activity among older adults. Considering the accumulated evidence of the health benefits of low-intensity physical activity, exploring and measuring such activities in this population is highly relevant. To strengthen strategic community actions enhancing health promotion and an active lifestyle, additional knowledge about physical activity patterns is necessary. Moreover, the visualization of physical activity can enable reflection on and awareness of activity habits, and preferences and facilitate behavior changes in older individuals.

## Figures and Tables

**Table 1:** The main categories for visualizing activities/physical activities in daily life. IEX: intentional exercise, METS: metabolic equivalents, NEPA: non-exercise physical activity, VISUAL-PA: software designed by the authors. The physical activity intensity level is represented by the following colors in VISUAL-PA: sedentary activity is red, light intensity NEPA is yellow, moderate/vigorous intensity NEPA is dark green, and intentional exercise of moderate/vigorous intensity is light green.

Daily Life	Main Category Label with Color Coding Used in VISUAL-PA	Examples of Activities	Examples of IEX	Examples of NEPA and their Intensity Level (METS) with Color Coding Used in VISUAL-PA	
**Physical activity patterns and sedentary behavior in daily life**	Self-care	Sleep, meals, personal hygiene	-	Sleep	Sedentary
				Get dressed, take a shower	Light
	Care for others	Help and foster others	-	Read for others	Sedentary
				Play with children (inside)	Light
				Play with children (outside)	Moderate/vigorous
	Household care	Keep home and possessions in order	-	Taking care of plants, iron	Light
				Vacuum, clean windows, garden work	Moderate/vigorous
	Recreation	Social activities, relations, reading, talking	-	Watching TV, reading	Sedentary
				Play music (own practice), painting	Light
	Transportation	Movement between places (active or passive mobility)	-	Taking the train/bus	Sedentary
				Taking dog out, bicycling, walking	Moderate/vigorous
	Food-related activities	Get ingredients, prepare food, and necessary activities afterwards	-	Shopping for food, baking, do the dishes by hand	Light
				Harvest from the forest, fish/hunt	Moderate/vigorous
	Intentional exercise (IEX)	Intentional exercise that includes structured, planned, and repetitive activities	Jogging, swimming, bicycling, walking, team exercise, resistance training	-	Moderate/vigorous
	Employed work/school	Work for income/education	-	Studying	Sedentary

## References

[1] Hamer M., de Oliveira C., Demakakos P. (2014). Non-exercise physical activity and survival: English longitudinal study of ageing. Am. J. Prev. Med..

[2] Lohne-Seiler H., Hansen B.H., Kolle E., Anderssen S.A. (2014). Accelerometer-determined physical activity and self-reported health in a population of older adults (65–85 years): A cross-sectional study. BMC Public Health.

[3] Svantesson U., Jones J., Wolbert K., Alricsson M. (2015). Impact of physical activity on the self-perceived quality of life in non-frail older adults. J. Clin. Med. Res..

[4] Barnett I., van Sluijs E.M., Ogilvie D. (2012). Physical activity and transitioning to retirement: A systematic review. Am. J. Prev. Med..

[5] Owen N. (2012). Sedentary Behavior: Understanding and influencing adults’ prolonged sitting time. Prev. Med..

[6] Dogra S., Ashe M.C., Biddle S.J., Brown W.J., Buman M.P., Chastin S., Gardiner P.A., Inoue S., Jefferis B.J., Oka K. (2017). Sedentary time in older men and women: An international consensus statement and research priorities. Br. J. Sports Med..

[7] (2015). Meld. St. 26 (2014–2015). Melding fra Storting om Fremtidens Primærhelsetjeneste. (The Primary Health and Care Services of Tomorrow—Localised and Integrated.

[8] (2013). Meld. St. 34 (2012–2013). Stortingsmelding om Folkehelse: God Helse—Felles Ansvar.

[9] Bouchard C., Blair S.N., Haskell W.L. (2012). Physical Activity and Health.

[10] Gabriel Pettee K.K., Morrow J.R., Woolsey A.L. (2012). Framework for physical activity as a complex and multidimensional behavior. J. Phys. Act. Health.

[11] Eckert K.G., Lange M.A. (2015). Comparison of physical activity questionnaires for the elderly with the International Classification of functioning, Disability and Health (ICF)—An analysis of content. BMC Public Health.

[12] Ekblom-Bak E., Ekblom B., Vikström M., de Faire U., Hellénius M.L. (2013). The importance of non-exercise physical activity for cardiovascular health and longevity. Br. J. Sports Med..

[13] Ip E.H., Church T., Marshall S.A., Zhang Q., Marsh A.P., Guralnik J., King A.C., Rejeski W.J., LIFE-P Study Investigators (2012). Physical activity increases gains in and prevents loss of physical function: Results from the lifestyle interventions and independence for elders pilot study. J. Gerontol. A Biol. Sci. Med. Sci..

[14] Amagasa S., Fukushima N., Kikuchi H., Takamiya T., Oka K., Inoue S. (2017). Light and sporadic physical activity overlooked by current guidelines makes older women more active than older men. Int. J. Behav. Nutr. Phys. Act..

[15] Sparling P.B., Howard B.J., Dunstan D.W., Owen N. (2015). Recommendations for physical activity in older adults. BMJ (Online).

[16] De Souto Barreto P. (2015). Global health agenda on non-communicable diseases: Has WHO set a smart goal for physical activity?. BMJ.

[17] De Souto Barreto P. (2015). Time to challenge public health guidelines on physical activity. Sports Med..

[18] Hübner L., Voelcker-Rehage C. (2017). Does physical activity benefit motor performance and learning of upper extremity tasks in older adults?—A systematic review. Eur. Rev. Aging Phys. Act..

[19] Arnadottir S. (2010). Physical Activity, Participation and Self-Rated Health among Older Community-Dwelling Icelanders: A Population-Based Study. Ph.D. Thesis.

[20] Scheers T., Philippaerts R., Lefevre J. (2012). Assessment of physical activity and inactivity in multiple domains of daily life: A comparison between a computerized questionnaire and the SenseWear Armband complemented with an electronic diary. IJBNPA.

[21] Bardach S.H., Schoenberg N.E., Howell B.M. (2016). What Motivates Older Adults to Improve Diet and Exercise Patterns?. J. Community Health.

[22] Orban K., Edberg A.K., Erlandsson L.K. (2012). Using a time-geographical diary method in order to facilitate reflections on changes in patterns of daily occupations. Scand. J. Occup. Ther..

[23] Hägerstrand T. (1970). What about people in regional science?. Papers in Regional Science.

[24] Ellegård K. (1999). A time-geographical approach to the study of everyday life of individuals—A challenge of complexity. GeoJournal.

[25] Jakobsen K. (2009). The right to work: Experiences of employees with rheumatism. J. Occup. Sci..

[26] Nilsson I., Blanchard M., Wicks A. (2013). Exploring everyday occupational engagement among community dwelling older people from a time-geographic perspective. Health Promot. Int..

[27] Vrotsou K. (2010). Everyday Mining: Exploring Sequences in Event-Based Data. Linköping Studies in Science and Technology. Ph.D. Thesis.

[28] Westermark Å. (2003). Informal Livelihoods: Women’s Biographies and Reflections about Everyday Life: A Time-Geographic Analysis in Urban Colombia. Ph.D. Thesis.

[29] Lester P.M. (2013). Visual Communication: Images with Messages.

[30] Palm J., Ellegård K. (2011). Visualizing energy consumption activities as a tool for developing effective policy. Int. J. Consum. Stud..

[31] Deyaert J., Harms T., Weenas D., Gershuny J., Glorieux I. (2017). Attaching metabolic expenditures to standard occupational classification systems: Perspectives from time-use research. BMC Public Health.

[32] Millward H., Spinney J.E., Scott D. (2014). Durations and domains of daily aerobic activity: Evidence from the 2010 canadian time-use survey. J. Phys. Act. Health.

[33] Tudor-Locke C., Leonardi C., Johnson W.D., Katzmarzyk P.T. (2011). Time spent in physical activity and sedentary behaviors on the working day: The American time use survey. J. Occup. Environ. Med..

[34] Van der Ploeg H.P., Merom D., Chau J.Y., Bittman M., Trost S.G., Bauman A.E. (2010). Advances in population surveillance for physical activity and sedentary behavior: Reliability and validity of time use surveys. Am. J. Epidemiol..

[35] Ainsworth B.E., Haskell W.L., Herrmann S.D., Meckes N., Bassett D.R., Tudor-Locke C., Greer J.L., Vezina J., Whitt-Glover M.C., Leon A.S. (2011). Compendium of Physical Activities: A second update of codes and MET values. Med. Sci. Sports Exerc..

[36] Bredland E.L., Magnus E., Vik K. (2015). Physical Activity Patterns in Older Men. Phys. Occup. Ther. Geriatr..

[37] Ellegård K., Vrotsou K. Capturing patterns of everyday life—Presentation of the visualization method VISUAL-TimePAcTS. Proceedings of the IATUR—XXVIII Annual Conference.

[38] Scheers T., Philippaerts R., Lefevre J. (2012). Variability in physical activity patterns as measured by the SenseWear Armband: How many days are needed?. Eur. J. Appl. Physiol..

[39] Vrotsou K., Ellegård K., Cooper M. (2009). Exploring time diaries using semi-automated activity pattern extraction. Electronic. eIJTUR.

[40] Sallis J.F., Cervero R.B., Ascher W., Henderson K.A., Kraft M.K., Kerr J. (2006). An ecological approach to creating active living communities. Annu. Rev. Public Health.

[41] Bauman A.E., Reis R.S., Sallis J.F., Wells J.C., Loos R.J., Martin B.W. (2012). Lancet Physical Activity Series Working Group. Correlates of physical activity: Why are some people physically active and others not?. Lancet.

[42] Bredland E.L., Söderström S., Vik K. (2018). Challenges and motivators to physical activity faced by retired men when ageing: A qualitative study. BMC Public Health.

[43] Loprinzi P.D., Lee H., Cardinal B.J. (2015). Evidence to support including lifestyle light-intensity recommendations in physical activity guidelines for older adults. Am. J. Health Promot..

[44] Jefferis B.J., Parsons T.J., Sartini C., Ash S., Lennon L.T., Wannamethee S.G., Lee I.-M., Whincup P.H. (2016). Does duration of physical activity bouts matter for adiposity and metabolic syndrome? A cross-sectional study of older British men. Int. J. Behav. Nutr. Phys. Act..

[45] Amagasa S., Machida M., Fukushima N., Kikuchi H., Takamiya T., Odagiri Y., Inoue S. (2018). Is objectively measured light-intensity physical activity associated with health outcomes after adjustment for moderate-to-vigorous physical activity in adults? A systematic review. IJBNPA.

[46] Powell K.E., Paluch A.E., Blair S.N. (2011). Physical activity for health: What kind? How much? How intense? On top of what?. Annu. Rev. Public Health.

[47] Palm J., Ellegård K. (2017). An analysis of everyday life activities and their consequences for energy use. Complex Systems and Social Practices in Energy Transitions.

[48] Van Cauwenberg J., De Bourdeaudhuij I., De Meester F., Van Dyck D., Salmon J., Clarys P., Deforche B. (2011). Relationship between the physical environment and physical activity in older adults: A systematic review. Health Place.

[49] Sørensen K., Van den Broucke S., Fullam J., Doyle G., Pelikan J., Slonska Z., Brand H. (2012). Health literacy and public health: A systematic review and integration of definitions and models. BMC Public Health.

